# Mucosal Herpes Immunity and Immunopathology to Ocular and Genital Herpes Simplex Virus Infections

**DOI:** 10.1155/2012/149135

**Published:** 2012-12-24

**Authors:** Aziz Alami Chentoufi, Lbachir BenMohamed

**Affiliations:** ^1^Pathology and Clinical Laboratory Medicine, Department of Immunology, King Fahad Medical City, P.O. Box 59046, Riyadh 11525, Saudi Arabia; ^2^Faculty of Medicine, King Fahad Medical City and King Saud Bin Abdulaziz University for Health Sciences, Riyadh 11426, Saudi Arabia; ^3^Laboratory of Cellular and Molecular Immunology, Gavin Herbert Eye Institute, School of Medicine, University of California, Irvine, Irvine, CA 92697, USA; ^4^Institute for Immunology, School of Medicine, University of California, Irvine, Irvine, CA 92697, USA

## Abstract

Herpes simplex viruses type 1 and type 2 (HSV-1 and HSV-2) are amongst the most common human infectious viral pathogens capable of causing serious clinical diseases at every stage of life, from fatal disseminated disease in newborns to cold sores genital ulcerations and blinding eye disease. Primary mucocutaneous infection with HSV-1 & HSV-2 is followed by a lifelong viral latency in the sensory ganglia. In the majority of cases, herpes infections are clinically asymptomatic. However, in symptomatic individuals, the latent HSV can spontaneously and frequently reactivate, reinfecting the muco-cutaneous surfaces and causing painful recurrent diseases. The innate and adaptive mucosal immunities to herpes infections and disease remain to be fully characterized. The understanding of innate and adaptive immune mechanisms operating at muco-cutaneous surfaces is fundamental to the design of next-generation herpes vaccines. In this paper, the phenotypic and functional properties of innate and adaptive mucosal immune cells, their role in antiherpes immunity, and immunopathology are reviewed. The progress and limitations in developing a safe and efficient mucosal herpes vaccine are discussed.

## 1. Introduction

Herpes simplex viruses types 1 and 2 (HSV-1 and HSV-2) are among the most common human infectious viral pathogens [1–3]. So many people have HSV-1 and/or HSV-2 but do not know that they have it [4, 5]. These two viruses can cause lifelong diseases with clinical manifestations including cold sores, genital ulcerations, corneal blindness, and encephalitis [6–8]. In cases of vertical transmission to the newborn, HSV-1 and HSV-2 can cause fatal neonatal encephalitis [9–11]. In the past two decades, there have been increasing reports of a worldwide pandemic of herpes infections despite the widespread use of antiviral drug therapies (reviewed in [12]). At the site of primary infection, HSV undergoes a productive replication within the epithelial cells lining the mucosa. Thereafter, the virus enters nearby sensory neurons, and the viral genome is transported to the neuronal nuclei in the sensory ganglia (trigeminal (TG) or dorsal root (DRG)) that innervate the infected site. During the first week after infection, HSV replication takes place in ganglionic sensory neurons, but within a few days no virus can be detected. While epithelial cells are destroyed during lytic HSV replication, most neuronal cells appear largely intact and serve as a reservoir for the latent virus. During reactivation, the virus travels from the TG and DRG back to the site of primary infection and causes eruptions on epithelial surfaces (viral shedding) with or without symptoms. This reactivation event may be spontaneous, but it is generally triggered by physical and chemical stress stimuli and/or with immunosuppression [7, 8].

### 1.1. Ocular Herpes

Herpes simplex virus type 1 (HSV-1) continues to spread around the globe. Ocular infection with HSV-1 is the leading cause of corneal blindness worldwide [7]. Following primary ocular infection, HSV-1 remains latent in the sensory neurons of trigeminal ganglia (TG) for the life of the host, with periodic stress-induced reactivation that produces progeny viruses in the eye causing potentially blinding recurrent corneal herpetic disease. Over 450 000 individuals in the USA have a history of ocular herpes. Ocular manifestations range from blepharitis and conjunctivitis to dendritic keratitis, causing disciform stromal edema and necrotizing stromal keratitis [7]. Antiviral drugs (e.g., acyclovir) reduce recurrent ocular disease by approximately 45% [13]. Because of the incomplete protection with these drugs [14–16], along with the emergence of acyclovir-resistant HSV strains [17–20], an efficient vaccine against HSV-1 and HSV-2, prophylactic or therapeutic, would be the most useful and cost-effective way to reduce morbidity and mortality [21–23].

### 1.2. Genital Herpes

Over 530 million worldwide are infected with HSV-2, a lifelong infection that continues to spread and can cause recurrent and painful genital lesions [1–3]. Recurrent genital herpes is the most prevalent sexually transmitted disease [24–26]. The Center of Disease Control and Prevention (CDC) reported in 2010 that (i) HSV-2 prevalence in the US remains high (16.2%) with women of all races at greater risk for HSV-2 infection and disease than men; (ii) the disease continues to disproportionately burden African Americans (39.2% prevalence), particularly black women (48.0% prevalence); and (iii)although less common as the cause of genitalherpes, there has been also a dramatic rise in the incidence of genital HSV-1 infections, mainly in young adults, largely due to the changes in sexual behavior. The percentage of primary genital herpes caused by HSV-1 has doubled during the last 2 decades, contributing to some 50% of all cases [7, 8]. While genital HSV-1 infections can result from genital-genital and oral-genital contact with an infected person who is actively shedding virus, oral-genital contact appears to account for most genital HSV-1 infections [7, 8]. Genital herpes has played a more important role than any other sexually transmitted infection in driving HIV prevalence [27]. Conversely, in HIV-infected individuals, HSV infection increases in frequency and severity as CD4 T-cell counts wane [27–30]. Patients with immunodeficiencies or immunosuppression-related treatments have an increased risk of developing severe HSV infection [31]. In the absence of strong local immunity, recurrent ulcerative lesions produced by reactivated HSV from sensory ganglia predispose and increase the risk of acquiring human immunodeficiency virus (HIV) [15, 32] and papillomavirus (PPV), which is associated with cervical carcinoma [33, 34]. Additionally, HSV infections can be fatal to newborns, of mothers that acquire the infection first time during pregnancy, and cause encephalitis or meningitis in adults [35]. A substantial number of HSV-2 seropositive individuals lack a history of clinically significant genital herpes [24, 25]. These asymptomatic individuals are the main source of virus transmission, which occurs mostly during periods of asymptomatic viral shedding [36, 37]. Despite the availability of many intervention strategies, such as behavioral education, condom use, and standard antiviral drug therapies, the transmission rate of herpes has continued to rise during the last three decades [38–40]. Although antiviral drug resistance has not been a major problem in immunocompetent patients, the problem of acyclovir resistance in immunocompromised patients is well documented [18, 41–47]. While genital herpes infection is wide-reaching, some populations are more affected despite the availability of condoms and chemoprophylaxis [48, 49]. Evidence suggests that only an effective vaccine against HSV might control this epidemic [49, 50]. However, the question that remains to be addressed is as follows: at which level the shedding has to be reduced by a vaccine in order to reduce transmission and disease?

### 1.3. Neonatal Herpes

Herpes affects some 30–60% of women receiving obstetric care, with newborns particularly susceptible to neonatal infection and severe herpetic disease [9, 51–53]. HSV-2 is responsible for up to 70% of neonatal herpetic infections [9–11, 51–53], which is defined as infection within 28 days of birth. However, HSV-1 appears also to cause more than 51% of neonatal herpes [9]. Seronegative women that contract the virus for the first time during pregnancy are at highest risk of transmitting the virus to the newborns [9, 11, 54]. HSV acquisition rates in pregnancy are high in discordant couples, especially for HSV-2 [55]. For neonatal transmission to occur, a pregnant woman must be shedding the virus at the time of delivery [9, 11, 54]. Approximately 85% of neonatal herpes results from the virus that is perinatally transmitted in the birth canal during the time of delivery [9, 51–53]. Studies of seropositive women have shown that HSV-2 is shed asymptomatically in the genital tract on approximately 1 of every 3 days [54, 56–61], a high proportion of which has significant implication on neonatal spread of HSV infections. Genital HSV-2 shedding at the time of delivery is associated with a relative risk of >300 for virus transmission. Neonatal herpes infection rates can be reduced by preventing maternal acquisition of genital HSV-1 and HSV-2 infection near term [62]. Perinatal and postnatal transmission may be prevented by the use of elective Caesarean delivery and avoidance of breastfeeding. The risk of neonatal herpes and death is highest in infants born to mothers who have seroconverted by the time of delivery. This implies a crucial role of the mother's immune system in minimizing vertical transmission. The prevalence and severity of neonatal HSV disease, as manifest by devastating CNS and disseminated infections, have increased over the past 10 years [51, 53, 63]. Prompt diagnostic testing of any mother/neonate at risk would be critical to protect the newborn. Once the newborn is infected, the most effective treatment for subsequent herpetic disease and alleviating potential neurodevelopment sequelae is oral acyclovir [9]. Once newborns develop neonatal herpes infection with central nervous system involvement, they are usually treated with parenteral or oral acyclovir (300–1500 mg per square meter) for 6 to 12 months [9]. Discontinuing this prophylaxis treatment in infants and young children with significant neurological sequelae sometimes leads to relapses even after 3 years of viral suppression. 

Neonatal vaccine is highly desirable, and the start point would be preventing the vertical transmission of the infection. The susceptibility of newborns to viral infections appears to be the consequence of the immaturity of their immune system [64]. Immunization of pregnant women with many other viral vaccines has been proposed and used successfully throughout the world for many years [65, 66]. Maternal vaccines for poliovirus, influenza viruses, and rubella have been found to be safe for both the mother and the newborn [66]. Since newborns are most susceptible to herpes infections but least responsive to vaccines [66], maternal immunization has been suggested as a way to protect newborns [67]. The benefit from maternal immunization may come from transferred immune antibodies across the placenta [68–71]. However, many questions still remain to be answered. (i) what would be the optimal level of maternal antibodies that are needed in order to prevent the transmission of the virus to newborn? (ii) How long will the transferred antibodies remain in the newborn at a magnitude that is high enough to prevent infection and disease? (iii) What is the relative role of innate immunity versus adaptive immunity in such protection? (iv) What is the relative role of HSV-specific antibodies in such protection?

## 2. The Mucosal Immune System and Herpes Immunity

 HSV-1 and HSV-2 infections occur at and emanate from mucosal surfaces. To combat herpes infection, the mucosal immune system maintains innate and adaptive immune barriers against these invading pathogens while avoiding overactive inflammatory responses that would impair mucosal tissue function. In human adults, the mucosal surface is enormous (up to 400 m^2^ of surface), with the mucosal immune system largely separate and distinct from the systemic immunity. In general, parenteral immunization induces systemic but not ocular mucosal immune responses, while ocular mucosal immunization induces both systemic and mucosal immune responses [72–74]. Within the common mucosal immune system, certain sites may facilitate a more far-reaching distal mucosal immune response than others, a sort of mucosal immune hierarchy. For example, antigens (Ags) administered intranasally promote immunity in the vaginal mucosa more effectively than Ags given orally, suggesting that there is compartmentalization or regionalization of the mucosal immune system. Intranasal immunization induces the production of IgA not only within the nasal cavity and salivary glands, but also in the small intestine lamina propria, the remote urinary tract, and the vagina. The vascular and lymphatic structures within the nasal mucosa as well as the nasolacrimal duct system provide unique anatomical conduits and intercommunication between the nasal-associated lymphoid tissue (NALT) and the ocular mucosal tissue, which are thought to be immunologically connected and interdependent. The integrated nature of OMIS and NALT systems is important for the development of ocular immunoprophylactic and immunotherapeutic vaccines, and it is hoped that intranasal immunizations will provide—or at least contribute to—ocular immune protection (and vice versa). Specifically in rats, topical ocular delivery of particulate antigen results in an Ag uptake that is greatest at the conjunctiva and an Ag uptake also in the NALT. In some cases, the induction site for Ag-specific IgA stimulation was traced to NALT rather than to the ocular surface. Therefore, it was suggested that NALT functions as a primary inductive site for ocular immune responses, at least in rodent models. However, this remains controversial and unresolved for humans where the complex interaction between OMIS and NALT is not yet fully elucidated.

 In order to better understand the immunity of herpes infections and ultimately design efficient therapeutic vaccines, it is fundamental to define the cellular and molecular immune mechanisms that control (or exacerbate) the infection/disease [7, 8]. It is important to note that, fortunately, only around 5% of immuno-competent HSV-infected individuals develop symptomatic herpetic recurrent disease (symptomatic individuals), while the majority of the infected human population remains asymptomatic despite continuously shedding from reactivated viral particles at a rate similar to symptomatic persons [7, 75–79]. Thus, while many have frequent recurrences of herpes disease (i.e., “symptomatic” or high-recurrent-disease patients with 1–5 episodes of recurrent disease/year), others have very few recurrences (i.e., “asymptomatic” or low-recurrent-disease patients with no history of recurrent disease). The difference between the symptomatic and asymptomatic groups is not a result of how often the latent herpes virus reactivates, as both groups shed the virus at similar rates [7, 77–79]. Instead, the difference is more likely related to variations in the magnitude and nature of cellular immune responses. As observed in animal models, herpes virus-specific T-cell responses have been reported to both protect against disease as well as cause disease [8]. It is not known why HSV-1 and HSV-2 reactivations/sheddings do not lead to symptoms in some individuals whilst it is symptomatic in others, or why the frequency and severity of recurrent disease vary among symptomatic individuals [8]. The immune mechanism(s) by which asymptomatic patients control herpetic infection and symptomatic patients do not remain to be fully elucidated [7, 75–79]. Identifying these mechanisms, or at least the viral antigens and epitopes involved, is critical to understanding how to protect against recurrent herpetic disease and for rational advances in therapeutic and/or prophylactic vaccine development. Until our recent studies [7, 75–79], little was known about the difference in T-cell responses in asymptomatic compared to symptomatic herpes patients.

 In this paper, T-cell determinants from herpes proteins that are recognized mostly by T cells from asymptomatic individuals are designated as “asymptomatic T cell epitopes”, while determinants that are recognized mostly by T-cells from symptomatic individuals are designated as “symptomatic epitopes.” A multitude of complex cellular and molecular mechanisms underlying the protective efficacy of T cells specific to “asymptomatic” epitopes versus the immunopathology of T cells specific to “symptomatic” epitopes may be in play [7, 77–79]. (1) The pathogenic “symptomatic” epitopes may direct the T-cell responses away from those that are best suited to clear the viral infection. (2) T-cell crossreactivity with epitopes from other viruses, within or outside the herpes family, can also play roles in protective heterologous immunity versus damaging heterologous immunopathology [79]. (3) The precursor frequency, proliferative capacity, and functional properties of epitope-specific “symptomatic” and “asymptomatic” T cells in a given individual may also be a factor [79]. Indeed, the T cell repertoire of individuals with the same MHC restriction elements can vary significantly based on “heterologous immunity” and “private specificity.” (4) “Asymptomatic epitopes” may trigger proliferation of “protective” T cells. Conversely, “symptomatic epitopes” may trigger proliferation of “pathogenic” T-cells [7]. Our recent findings support different levels of HSV-specific T-cell repertoires in symptomatic and asymptomatic individuals [7, 77–79]. We found that T cells from symptomatic and asymptomatic individuals, with similar HLA, have dramatically different profiles of responses to HSV epitopes. A set of human T-cell epitopes from HSV-1 glycoproteins B and D (gB and gD) is strongly recognized by T cells from HSV-1 seropositive asymptomatic individuals, but only weakly by T cells from symptomatic individuals [7, 77–79]. We recently made the unique observations that following. (1) A set of “promiscuous” human HSV-1 and HSV-2 glycoprotein B (gB) epitopes was strongly recognized by T effector cells (T_eff_ cells) from asymptomatic patients but not by T cells from symptomatic patients [1]. In contrast, a different nonoverlapping set of gB epitopes was strongly recognized by T-cells from symptomatic patients, but not by T cells from asymptomatic patients [7, 79]. (2) More importantly, immunization of susceptible double transgenic mice, expressing both type 1 and type 2 human leukocyte antigens (i.e., HLA-DR and HLA-A2.1) with “asymptomatic” T-cell epitopes reduced the severity of herpetic lesions when inoculated with HSV-1 and HSV-2 (submitted). We, therefore, hypothesize that different sets of HSV-1 and HSV-2 T-cell epitopes are recognized by symptomatic versus asymptomatic individuals and that protective immunity against herpes disease can be induced following immunization with “asymptomatic epitopes,” but not “symptomatic epitopes.”

### 2.1. Ocular Mucosal Immune System (OMIS) and Ocular Herpes Immunity

The ocular mucosal surface is the first-line defense system that is frequently exposed to infections [80]. The conjunctiva and the lacrimal glands are the key components of ocular mucosal immune system (OMIS) (reviewed in [81]). Topical ocular immunization—rather than parenteral immunization—is most likely to induce critical local mucosal immune responses [23, 82, 83]. We recently observed that topical ocular immunization (eye drops) with lipopeptides (peptides linked to fatty acid moiety) was more effective at inducing ocular mucosal immune responses than parenteral immunization (unpublished). Interestingly, intranasal immunization (nose drops) was even more effective than topical ocular immunization (possibly because of better retention of the inoculum). Since lipopeptide vaccines bearing murine herpes CD4^+^ and CD8^+^ T-cell epitopes (unpublished and [84–86]) are able to cross ocular and nasal surfaces, we hypothesize that herpes lipopeptides bearing human T-cell epitopes will also be able to cross the mucosal membrane and deliver the specific epitopes to both the local ocular and systemic immune systems.

 Although many investigators have studied mucosal lymphoid sites of the common mucosal system, the focus has been mostly on gut-associated lymphoid tissue (GALT), nasal associated lymphoid tissue (NALT), and vaginal-associated lymphoid tissue (VALT). Only a small number of researchers are actively involved in studying OMIS, also known as eye-associated lymphoid tissue (EALT) [23]. As mentioned before, among the unanswered questions in OMIS biology is the role of the NALT in generating OMIS immunological protection and vice versa. The conjunctiva and the lacrimal glands are key elements in the OMIS [23]. The conjunctiva forms a continuous mucosal surface that extends from the eyelid margin to the cornea and makes contact with airborne pathogens and periocular tear film [23]. The conjunctiva and the lacrimal gland are postulated to play an active role in both inductive and effector functions with the presence of IgA+ plasma cells, secretory IgA (sIgA), and immune cells that produce cytokines and chemokines [81, 87–91]. Conjunctival immuno-competent cells include those from the lymphoid system (lymphocytes) and those from the myeloid system such as macrophages, polymononuclear leukocytes, eosinophils, mast cells, basophils, fibroblasts, epithelial cells, vascular endothelial cells, and classical antigen presenting cells (macrophage, dendritic cells, Langerhans cells, and B cells). Human conjunctiva contains an abundance of lymphoid-derived cells [23]. Most (92%) of the conjunctiva's lymphocytes are T cells (76% are CD8^+^ T cells, and 16% are CD4^+^ T cells; [23] memory CD45(+)RO(+) T cells constitute 45% of total CD45(+) leukocytes, while naive CD45+RA+ T cells represent 29%) [23]. We have recently demonstrated that OMIS CD4^+^ T cells and serum IgA can be induced in rabbits following topical ocular delivery of HSV peptides together with cytosine-phosphate-guanine (CpG_2007_) mucosal adjuvant [83], a TLR-9 ligand. Like other components of the mucosal system, ocular mucosal immune system (OMIS) maintains a barrier against exogenous antigens and invading infectious pathogens that attack the surface of the eye and GT while avoiding inflammatory responses that would impair parenchymal function. T lymphocytes, both CD4^+^ and CD8^+^ phenotypes, and plasma B cells together account for more than 60% of the entire mucosal effector immune system cell population.

Recently, we have described an abundance of “natural” Foxp3+CD4+CD25+ nT_reg_ cells in rabbit conjunctiva, the main inductive site of the ocular mucosal immune system [76]. We demonstrated that conjunctiva-resident nT_reg_ cells suppress HSV-1-specific CD4+ and CD8+ effector T cells (T_eff_). Converging evidence from our laboratory and other laboratories demonstrates that nT_reg_ cells have the potential to dampen the vaccine-induced, HSV-specific, T_eff_-mediated immunity [76]. Despite recent extensive studies on nT_reg_ cells, the molecular mechanism by which nT_reg_ cells mediate the suppression of pathogen-specific T-cell immunity or dampen vaccine-induced T_eff_ cells remains poorly understood.

To prevent excessive immune-mediated ocular tissue damage by T_eff_ cells, a proper balance between the T_eff_ cell, and T_reg_ cells is needed. This balance was thought to be maintained by communication through cytokines or by stimulation through costimulatory molecules on APCs [76]. However, it was recently shown that TLRs in mouse and human T_reg_ cells sense pathogens directly and modify T_reg_ action [81, 87–91]. TLRs ligands, such as CpG, can also modulate immune responses by blocking the suppressive effects of T_reg_ cells. The TLR profile and function of conjunctiva T_reg_ cells have not been reported. In order to study the role of T_reg_ in ocular herpes immunity, we recently examined the expression profile of TLRs on conjunctiva-resident nT_reg_ cells and assessed whether TLRs are functionally active by stimulating these nT_reg_ cells with TLR agonists in the absence of APCs. We have shown that rabbit conjunctiva-resident CD4^(+)^CD25^(+)^  nT_reg_ cells express high levels of functional TLR2 and TLR9. Topical ocular immunization of rabbits with HSV-gD peptide T-cell epitopes, together with a TLR2 ligand (LTA), reverses CD4^(+)^CD25^(+)^  nT_reg_ cell suppressive function. In contrast, topical ocular immunization of rabbits with the same epitopes delivered with a TLR9 ligand (CpG_2007_) resulted in only a slight effect on CD4^(+)^CD25^(+)^nT_reg_ cell suppressive function. Our findings demonstrate that regulating conjunctiva CD4^(+)^CD25^(+)^  nT_reg_ cell function trough TLR2 and TLR9 leads in turn to the modulation of ocular mucosal HSV-specific CD4^(+)^CD25^(−)^  T_eff_ cell responses. 

 Within the common mucosal immune system, certain sites may facilitate a more far-reaching distal mucosal immune response than others [92, 93]. For example, antigens administered intranasally promote vaginal immunity more effectively than antigens given orally, suggesting that there is compartmentalization or regionalization of the mucosal immune system [34, 92]. Intranasal immunization induces IgA not only within the nose and salivary glands, but also in the small intestine lamina propria [94], the remote urinary tract [95], and the vagina [96]. The vascular and lymphatic structure of OMIS and nasolacrimal duct system provide unique anatomical conduits through which the NALT (nasal-associated lymphoid tissue) and OMIS are thought to be immunologically connected and interdependent. The integrated nature of OMIS and NALT systems is important for ocular immunoprophylactic and immunotherapeutic vaccines considerations, and it is hoped that intranasal immunizations will provide—or at least contribute to—the ocular immune protection (and vice versa). In rats, following a topical ocular delivery of particulate antigen, the antigen uptake is greatest at the ocular sites, particularly the conjunctiva, and also in NALT [81–83, 91, 97]. In some cases, the induction site for antigen-specific IgA stimulation was traced to NALT rather than the ocular surface [98]. Therefore, it was suggested that NALT functions as a primary inductive site for ocular immune responses, at least in rodent models [98]. 

 Recently, by using a simple surgical procedure in rabbits, we disconnected the OMIS from NALT and assessed the immunogenicity of a vaccine formulation administered either ocularly or intranasally [99]. We showed that NALT do interact immunologically with the OMIS through the nasolacrimal ducts. Topical ocular immunization-induced T-cell responses in the conjunctiva did not appear to be modulated by NALT [99]. However, NALT appeared to downmodulate systemic immune responses [99]. Conversely, nasal immunization efficiently induced conjunctival T-cell responses. The mechanisms by which NALT downmodulated ocular mucosal immune responses induced following topical ocular immunization remain to be identified. It is possible that the nature of the immune response induced by NALT during topical ocular immunization could generate suppressive cells or factors that downmodulate the systemic Th1 immune response [99]. 

**Figure 1:**
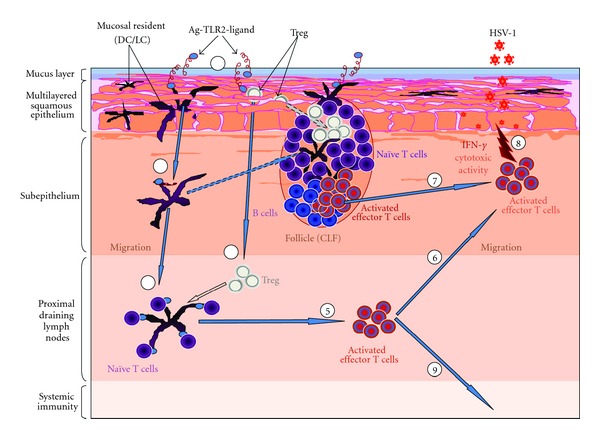
Model of immune mechanisms of TLR2-mediated control of an anti-HSV-1 immune response in the conjunctiva. During topical ocular immunization with TLR2 agonist (1), high concentration of exogenous TLR2 agonist is sensed by conjunctiva APC and Treg cells residing in the epithelium and then triggers the migration of conjunctiva resident APC (DC) and Treg to the proximal draining lymph node (2, 4) or (dashed arrow) to the conjunctiva lymphoid follicles (CLF). TLR2/TLR2L interaction promotes maturation of DC and proliferation of Tregs paralleled by temporarily abrogated suppression (empty arrow). As a result, Tregs do not suppress the ongoing immune response in the draining lymph node or in CLF. (3) DC stimulate naïve CD4+ and CD8+ effector T cells which undergo cell division and proliferation in the draining LN (5) or CLF where they form with B cells a germinal center (characteristic of lymphoid follicles). Activated effector T cells migrate to the epithelium and kill HSV-1-infected epithelial cells through CTL activity. Some activated effector cells in the LN preferentially migrate to the periphery through lymphoid afferent canal to induce systemic immune response. Once HSV-1 is cleared by the immune system and the source of TLR2 ligands is no longer present, Tregs will regain their suppressive capabilities, thus contributing to the balance between tolerance and immunity.

 Conjunctival lymphoid follicles (CLFs) undergo hyperplasia in the presence of a pathogenic infection (e.g., HSV-1), and CLFs appear to participate in the afferent limb of the acquired immune responses for the ocular surface [23]. The presence of plasma cells in human conjunctiva suggests efferent function as well with the expression of secretory component by human conjunctival epithelium. The function of human conjunctival lymphoid follicles is still debated [23]. Human conjunctival B cells may be induced to differentiate into plasmocytes secreting sIgA following pathogen or Ag stimulation. Similar to humans, rabbit conjunctiva contains an abundance of CLFs [23]. Conjunctival lymphoid follicles (CLFs) exist in normal individuals as organized sub-epithelial collections of lymphoid cells, often with germinal centers [23]. The human conjunctiva and CLF are part of OMIS and participate in afferent adaptive ocular immunity. CLFs are also found in rabbit conjunctiva but are absent in mice and rats [23].  Figure 1 describes the mechanisms of a vaccine-mediated control of an anti-HSV-1 immune response in the conjunctiva following ocular infection with HSV-1.

### 2.2. Genital Tract Mucosal Immune System and Herpes Immunity

Mucosal genital surfaces represent the entry point of both HSV-1 and HSV-2, neither of which has an effective vaccine. Genital herpes is an incurable, widespread sexually transmitted disease that continues to contribute significantly to morbidity and mortality worldwide [2], particularly in neonates and immunocompromised individuals [2]. In women, herpes simplex virus type 2 (HSV-2) infects the mucosa in the genital tract and spreads to the nervous system. After the initial infection is resolved, latent virus can persist in infected ganglia for long periods, and the activation of the latent virus causes recurrent disease [2]. Maintenance of the integrity of the epithelium of the genital tract is critical for sexual and reproductive health. It serves as a physical barrier to protect the host against infection without compromising critical reproductive functions [2]. The vagina, which is populated by commensal microflora, is lined with stratified squamous epithelial cells, while the uterus and Fallopian tubes are lined with columnar epithelium. The female sex hormones, estradiol and progesterone, influence and regulate epithelial cell function and barrier integrity throughout the reproductive tract [2]. In addition to acting as a physical barrier, the epithelium functions as an integral part of the innate and adaptive immune systems. 

 Protection against potential pathogens in the female genital tract (GT) is provided by a variety of measures that can be grouped into two broad categories: innate and adaptive immunities. The GT mucosa is unique in the regulation of immune protection as it is exposed to sexually transmitted bacterial and viral pathogens, allogeneic spermatozoa, and the immunologically distinct fetus [2]. In response, GT has evolved immune mechanisms to protect against pathogens without compromising fetal survival. While much attention has been paid to innate immune function in the lungs and GI tract [2], very few studies have investigated the presence and function of the innate immune system in the GT. It has become clearer that the innate immune system is present throughout the reproductive tract and functions in synchrony with the adaptive immune system to provide optimal protection. 

 Similar to CLF (Figure 1), an induced vaginal-associated lymphoid tissue aggregate has been documented in the genital mucosa of immunized mice, which has been correlated with protection against HSV-2 infection following the challenge [2]. These aggregates contain CD4^+^ T cells, B cells, and CD11c+ antigen-presenting cells. Furthermore, it has been suggested that the local microenvironment in genital tract plays a role in generating effective antiviral immune responses after immunization. Zhang et al. [2] have examined whether protective effector memory T-cell responses could be induced in the genital mucosa in the absence of secondary lymphoid organs, utilizing a lymphotoxin knockout mouse model. Intravaginal (IVAG) immunization of both lymphotoxin^(−/−)^ and parental wild-type (WT) mice lead to complete protection against genital HSV-2 challenge. The immune responses generated were effective in protecting against mucosal HSV-2 challenge in the genital mucosa, suggesting that even in the absence of secondary lymphoid organs, IVAG immunization could induce an effective anti-HSV-2 memory T cell response. This suggests the importance of tissue-resident effector memory T cells in the protection against genital herpes as recently reported [100–102].

 Several lines of evidence, in both animal model [8] and humans [7], support a critical role for CD8+ T cells in the control of HSV-2 infections and in surveillance function that limits reactivation from sensory ganglia and muco-cutaneous tissues. The precursor frequency of HSV-2-specific CD8+ CTL is correlated with HSV-2 severity in HIV-1/HSV-2 coinfected men [2] and cross-sectional studies show HLA class I associations with HSV severity [7, 8]. Local infiltration of HSV-2-specific CD8^+^ CTL correlates with clearance of virus particles in human recurrent herpes [7, 8]. HSV-2-specific CD8+ T cells persistently infiltrate healed genital herpes lesions and localize near sensory nerve endings [7, 8]. How local HSV-2-specific CD8+ T cells in dorsal root ganglia interact with infected neurons remains to be determined. In mice, HSV-2-specific CD8+ T cells infiltrate infected ganglia during the acute and latent phase, and mediate control over viral reactivation in an IFN-*γ*-dependent manner [7, 8]. In genital biopsy specimens from humans with recurrent HSV-2 infection, viral clearance is associated with a high concentration of local CD8+ T cells with cytolytic activity against infected cells [7, 8]. In animal models, depletion of CD8^+^ T cells impairs clearance of virus from sensory neurons, whereas TCR transgenic CD8^+^ T cells specific for the immune-dominant H-2K^b^-restricted peptide in HSV-2 glycoprotein B (gB_498–505_) transferred into mice lacking other components of adaptive immunity result in viral clearance [8]. These studies demonstrate that HSV-2-specific CD8+ T cells play a protective role in HSV-2 infection.

## 3. CD8^+^ T-cell Functions during HSV-1 and HSV-2 Latency/Reactivation Cycle 

**Figure 2:**
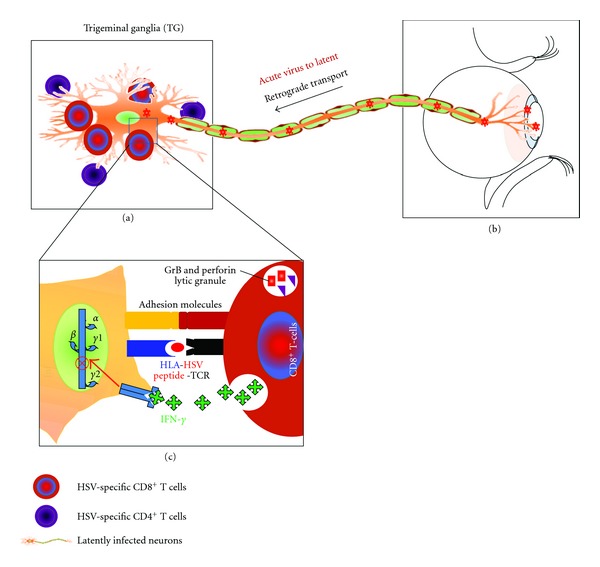
A model of CD8^+^ T-cell monitoring of HSV-1 latency in sensory ganglia. (a) During a primary infection (affecting the cornea of the eye), HSV-1 invades the termini of sensory neurons, the nucleocapsid travels by retrograde axonal transport to the neuron cell bodies within the trigeminal ganglion (TG), viral DNA is inserted into the nucleus, and a brief period of virus replication ensues (b). An initial infiltration of macrophages and T cells gives rise to an infiltrate dominated by CD4+ and CD8+ T cells and macrophages that persists for the life of the animal (c). The CD8+ T cells associate closely with the neuron cell bodies and directly monitor viral gene expression in neurons by detecting epitopes of viral epitopes that are produced early in a reactivation event and presented on the surface of the neuron within MHC class I. The CD8+ T cells force the viral genome into a quiescent state through IFN-*γ* production (early in reactivation) or through the release of lytic granules (at later stages of reactivation). A similar model can be extrapolated to genital herpes infection with HSV-2.

Following primary ocular infection in immune-competent humans, HSV-1 and HSV-2 establish a lifelong latent infection in neurons of the sensory ganglia (SG) with intermittent reactivation cycles (Figure 2). While spontaneous reactivation of the virus from sensory ganglia and shedding of the virus in tears or in genital tract do not seem to occur in mice (as opposed to rabbits, guinea pigs, and humans), reactivation of HSV from latent infection is readily observed in vitro when sensory ganglia are explanted in culture [103]. HSV reactivation in mice can be induced to a limited extent by ultraviolet irradiation [104] or elevated body temperature or hormone [105, 106]. A recent study used a restraint “stress” mouse model of virus reactivation [107]. In this model, mice are subjected to stress by restraining them in aerated plastic tubes for 12 hrs and depriving them of food and water for the same period of time. Obviously, this protocol of extreme and acute stress does not represent the daily “physiological” stress situations in humans (be that a physical or chemical stress). Exposure of HSV latently infected host to stress may upregulate certain molecules, compromising the efficacy of protective CD8^+^ T-cell response, and may also diminish the TG-resident CD8^+^ T-cell population [108–111], thus inducing HSV reactivation from latency [112].

**Figure 3:**
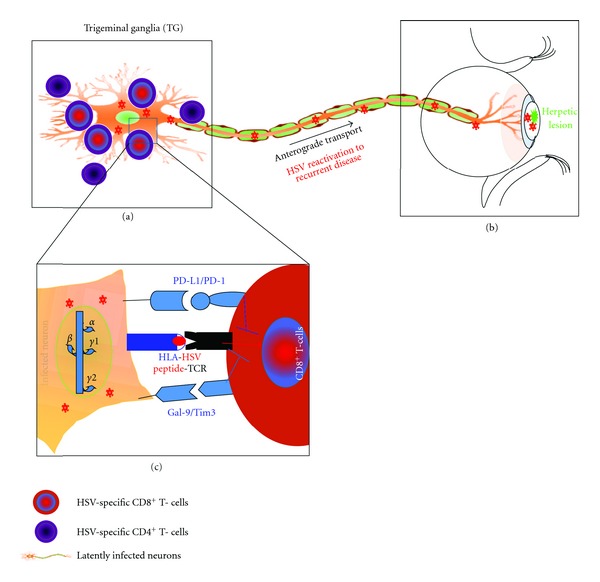
A model of CD8^+^ T-cell exhaustion and HSV-1 reactivation from sensory ganglia. During reactivation, the virus travels from the TG back to the cornea and causes eruptions of epithelial surfaces (viral shedding). Viral reactivation may be asymptomatic or may be associated with symptoms or lesions [7, 76–79]. This reactivation event may be spontaneous, but it is generally believed to be triggered by stress stimuli and immunosuppressive conditions. CD8+ T-cell function is compromised (e.g., by stress-related hormones), viral glycoproteins and nucleocapsids are formed and transported by anterograde axonal transport, virions are assembled at nerve termini, and infectious virus is released, potentially leading to recurrent disease. Evidence from our laboratory and others suggested that latently infected neurons appear to be resistant to CD8+ T-cell-mediated killing and that LAT is involved in this resistance to CD8+ T-cell killing. Recently, we found that neuroblastoma cells expressing LAT in the absence of other HSV-1 genes resisted to GrB-induced apoptosis and are protected from CD8+ T-cells attack. Also, latently infected TG have high expression of PDL-1 and Gal9 on infected neuronal cells also the majority of CD8+ T-cells surrounding neuronal cells express high PD-1 and Tim3.

**Figure 4:**
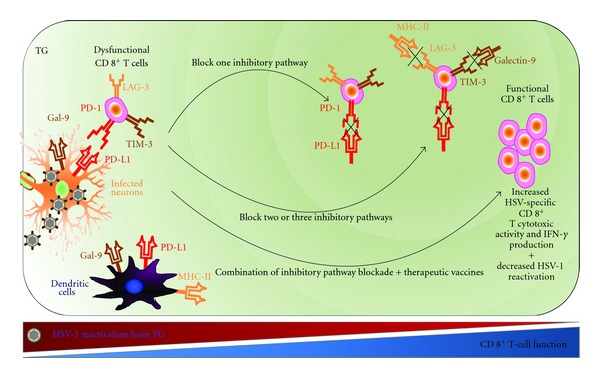
Blockade of T-cell inhibitory pathways to boost immunity to herpes simplex virus infections. Multiple inhibitory pathways may be activated in the exhausted CD8+ T cells in the HSV-1 latently infected TG, including PD-1, TIM-3, and LAG-3. Blockade of one T-cell inhibitory pathway may partially restore HSV-specific CD8+ T-cell effector functions. Blocking antibodies may be directed against the PD-1, TIM-3 and LAG-3 T-cell inhibitory receptors on CD8+ T cells or possibly their ligands (PD-L1 galectin-9 and MHC-II, resp.) on infected neurons or on DC. Full restoration of CD8+ T-cell function may require blockade of two or more inhibitory pathways or a combination of pathway blockade and vaccination. Sustained restoration of DC maturation may be also crucial for functional CD8+ T-cell function and clinical cure. HSV-1 LAT gene appears-interfering with both CD8+ T-cell function and with DC maturation. How CD8+ T cells are dysfunctional and DC are silenced, and the pathways to rescue this silencing, is still unknown.

Herpes latency in human SG is accompanied by a chronic CD8^+^ T-cell infiltrates that suppress virus reactivation from latency [113, 114]. However, even in the face of local CD8^+^ T-cell responses, HSV-1 and HSV-2-still periodically reactivates from the SG, travel back to the mucocutaneous surfaces via sensory neurons, and can cause potentially blinding recurrent herpetic disease [115–117]. This suggests that HSV-1 and HSV-2 have evolved mechanisms to evade CD8^+^ T-cell immunosurveillance [115–117]. However, the exact cellular and molecular mechanisms by which HSV-1 and HSV-2 intermittently escape CD8^+^ T-cells immunosurveillance are unknown. Latency-associated transcript of (LAT) HSV-1 and HSV-2 is the only viral gene that is abundantly transcribed in latently infected SG. LAT is essential for efficient spontaneous reactivation and promotes neuronal survival by reducing apoptosis [118–120]. LAT can be considered as an immune evasion gene, since we have recently found that, (1) LAT inhibits Granzyme-B-(GrB-) mediated CD8^+^ T cell killing by blocking GrB-induced apoptosis [121]; (2) LAT increases PD-1, TIM-3, and LAG-3 on TG resident CD8^+^ T cells and promotes inhibition of HSV-specific CD8^+^ T-cell function in latently infected TG, which is consistent with the known higher reactivation of LAT^(+)^ versus LAT^(-)^ virus [6, 87, 114, 121, 122] (Figure 3); (3) LAT increases MHC and PD-L1 on neuroblastoma cells in vitro and in total TG extracts in vivo ([87, 113, 114] and Appendix); and finally, (4) LAT increases GAL-9, the ligand of TIM-3, in total TG extracts in vivo (not shown). Based on these results, together with related reports by others in the field [115–117, 123, 124], we hypothesize the following. (1) Interactions between PD-1^+^CD8^+^ T cells, TIM-3^+^CD8^+^ T cells and/or LAG-3^+^CD8^+^ T cells in the TG with PD-L1, GAL-9, and/or MHC-II expressing LAT^(+)^ neurons result in inhibition of HSV-specific CD8^+^ T-cell function and impaired anti-HSV immunity. As illustrated in Figure 4, blocking the T-cell inhibitory pathways, combined with therapeutic vaccination, may restore the function of HSV-specific CD8^+^ T cells in TG and reduce virus reactivation. The investigation of these combined therapies may open new doors to novel T-cell-based interventions to reduce/stop HSV-1 and HSV-2 reactivation and prevent recurrent disease.

Dysfunctional CD8^+^ T cells display impaired effector function that is characterized by decreased production of proinflammatory cytokines and hyporesponsiveness to antigenic restimulation. Total or partial loss of T-cell function (dysfunction) occurs during many latent and chronic infections [125], including latent HSV-1 infection [113, 123, 126]. However, not all T-cell dysfunction is due to exhaustion. Exhaustion is usually linked with expression of PD-1 and TIM-3, while dysfunction is linked with one or more of the eight T-cell inhibitory receptors described previously, which include PD-1 and TIM-3. T-cell dysfunction requires two signals: a first signal through the T-cell receptor (TCR) following epitope presentation to TCR via the MHC complex [127]; and a second signal through costimulation from T-cell inhibitory receptors. In humans*:* latent HSV-1 in human TG is accompanied by a chronic CD8^+^ T-cell infiltration [128]. At least a portion of viral latency/reactivation in human TG appears to be controlled by CD8^+^ T cell-mediated mechanisms [87, 113, 114]. Significant numbers of CD8^+^ T cells producing IFN-*γ* were found in latently infected TG of human cadavers, suggesting an antigen-driven T-cell response [129–132]. However, many TG-resident CD8^+^ cells express PD-1 and appear to be dysfunctional [133]. In mice, similar to what is seen in humans, latently infected TGs have a chronic CD8^+^T-cell infiltration. CD8^+^ T cells accumulate in TG from 7 to 10 days following ocular herpes infection and become the predominant T-cell type during latency [134]. HSV-specific CD8^+^ T cells producing IFN-*γ* and GrB appear to suppress (or abort) induced viral reactivation in explanted mouse sensory ganglia [134, 135] and may similarly reduce detectable HSV-1 reactivation in vivo [136–139]. We have found dysfunctional CD8^+^ T cells specific to human epitopes in the LAT^(+)^ TG in the “humanized” HLA Tg mice. A human therapeutic vaccine that increases the size and functionality of the HSV-specific IFN-*γ* ^+^GrB^+^CD8^+^ T-cell population in latently infected TG should significantly decrease the rate of spontaneous reactivation (as measured by shedding in tears) and reduce recurrent eye disease. During neuronal latency, high levels of HSV-1 LAT RNA can be readily and consistently detected in the TG [140, 141]. HSV-1 LAT null mutants (LAT^(−)^) generally have a reduced reactivation phenotype [142–144], indicating that LAT plays an important role in the HSV-1 latency-reactivation cycle. LAT can block apoptosis [120], which supports wild-type reactivation [120].

## 4. Topical Mucosal Vaccines to Stimulate Herpes-Specific Mucosal Immunity

Since the 1920s, there have been countless research efforts for the development of a herpes vaccine [145–148]. Eight decades later, while important research gains have been made, no clinically approved vaccine is yet available [145–149]. In the past, numerous vaccine approaches using live attenuated virus, killed virus, or recombinant protein-based vaccines products showed efficacy in animal models but failed in clinical trials [122, 150–152]. The majority of the vaccines are injected parenterally. While they induced strong systemic immune responses, they failed to generate sufficient local immune responses either in the eye, TG, or draining lymph nodes, which are likely needed to prevent virus transmission and to reduce replication [23, 84, 85, 150, 153–160]. The challenge in herpes vaccine development is to induce higher magnitude and wider breadth of the immune response [122, 150, 161–163]. However, the progress towards a herpes vaccine still faces many challenges, among which are (1) the identification and inclusion in the vaccine of critical “protective” epitopes recognized by asymptomatic patients; (2) the exclusion of potentially “pathogenic” epitopes recognized by symptomatic patients; and (3) the optimization of an efficient and safe mucosal vaccine delivery system [21, 23, 82–86, 146].

### 4.1. Ocular Mucosal Herpes Vaccines

The viral epitopes involved in protective versus pathogenic immune responses are critical for a rational design of an epitope-based ocular herpes vaccine [37, 164–166]. Considering the wealth of data addressing the role of T cells in animal models, it is surprising how little is known about the nature and magnitude of HSV-specific T-cell responses in asymptomatic versus symptomatic patients. Therefore, we have started to examine the asymptomatic and symptomatic patients' T-cell responses against a library of potential epitopes identified from gD and gB [1]. 

Unmodified synthetic peptides usually fail to prime T-cell responses in vivo, unless they are delivered with a potent immunological adjuvant [167–169]. Peptide-based T-cell epitopes have been emulsified with a variety of adjuvants, including Freund's [167–170], Montanide's ISA-51 and ISA-720 [73, 74, 151, 171–173], MF59 [174–176], and QS-21 [177]. Most of these adjuvants tested in small laboratory animals have limitations due to toxicity. Others fail to reproduce in humans the results obtained in mice (reviewed in [178]). Recently, lipopeptide-based vaccines (i.e., peptides covalently linked to a fatty acid moiety) have gained considerable interest and represent a promising approach for vaccine delivery [73, 74, 151, 171–173, 179–192]. We and others have shown that parenteral or mucosal administration of lipopeptide immunogens from HIV, HBV, HCV, HPV, CMV, HSV, group A streptococcus, and Plasmodium *falciparum* malaria, without external adjuvant, is efficient in inducing both local and systemic protective CD4^+^ T helper and CD8^+^ T-cell responses [73, 74, 151, 171–173, 179–182, 185–193]. Lipopeptide Ags are taken up by mucosal dendritic cells/Langerhans cells (DC/LC), inducing phenotypic maturation of DCs, which are then capable of priming T cells at the systemic and mucosal levels [73, 74, 151, 171–173, 179–192]. In nonhuman primates, we showed that lipopeptide vaccine provided strong protection against malaria [172, 173, 194]. At the time this is being written, several clinical trials using malaria and HIV lipopeptides are being conducted in Europe. In a recent phase I clinical trial, both intradermal and intramuscular immunizations with an HIV lipopeptide vaccine induced strong T-cell immune responses [195]. This first wave of preclinical and clinical trials showed that lipopeptide-based vaccines are efficient, safe, and can be manufactured in large scale at GMP levels by modern techniques of chemoselective ligation [183, 184]. Recently, we found that immunization with a cocktail of three immunodominant CD4^+^ T-cell lipopeptides from gD induced more efficacious protection against ocular herpes infection and disease than any single lipopeptide alone [84]. This finding strongly suggests that multiple epitopes can induce a robust T-cell-mediated protective immunity against ocular herpes [84, 85]. This is probably due to the generation of more CD4^+^ and CD8^+^ T-cell responses against multiple epitopes resulting in polyclonal T-cell lines (one T-cell clone for each epitope). Efforts in designing peptide immunogens for the induction of multiple HTL and CTL responses included various strategies such as multiple Ag peptide (MAP) conjugates [196, 197] and sequential arrangement of epitopes into a single polypeptide [198, 199]. Multiple antigenic peptide constructs have been shown to be potent, but have been challenging to be produced in large quantities. Linear polypeptides are more efficient than MAP [196, 197] and can be produced by standard techniques of peptide synthesis. In addition, to avoid any potential junctional epitopes that may be created by adjacent epitopes, each epitope is separated with a GPGPG spacer [196].

### 4.2. Genital Herpes Mucosal Vaccines

In most of the clinical trials, the vaccines failed to protect from infection in spite of inducing strong HSV-specific neutralizing antibody responses, emphasizing a crucial role for cell-mediated immunity, especially on type 1 immunity [145–148]. While important research gains have been made, there is still no clinically approved vaccine for the prevention or treatment of herpes infection and diseases. The challenge in herpes vaccine development is to induce a higher degree and breadth of T-cell responses [122, 150, 161–163]. In addition, the majority of genital herpes vaccines are delivered parenterally and do not generate significant mucosal T-cell immunity neither (i) at the site of infection nor (ii) in the local lymph nodes that drain the genital tract (GT). T-cell immunity at both sites is likely necessary to prevent transmission and limit severity of genital herpes [154–160]. Furthermore, subunit formulations delivered into the GT are poorly immunogenic compared to other mucosal routes (e.g., intranasal route) [154, 200–202]. The progress towards an intravaginal (IVAG) T-cell-mediated vaccine still faces significant challenges, among which are (1) the identification of critical human “protective” CD4^+^and CD8^+^  T_eff_ cell epitopes (i.e., epitopes mostly recognized by T cells from asymptomatic patients); (2) the improvement of protective “naturally processed” T_eff_ cell epitopes; and (3) an efficient and safe IVAG immunization strategy [21, 23, 82–86, 146, 147, 203, 204]. 

## 5. Mucosal Herpes Immunopathology

A study recently compared HSV-1 infection to HSV-2 infection in two different mucosal sites (ocular and genital sites) in the mouse model demonstrated that despite the elevated chemokines and cellular responses to HSV-2 in the cornea, vagina, BS, spinal cord, and lymph nodes, HSV-2 still replicates at a greater rate than HSV-1 in the genital mucosa and presents higher viral titers in the TG as well [205]. However, this study must be extended to different strains of HSV-1 and HSV-2 and maybe also to some clinical HSV-1 and HSV-2 isolates. Whether the finding accounts for the extent of the disease caused by these closely related alphaherpesviruses and in each mucosal site remains to be determined. Unknown also is the relative extent of virus replication and immunopathology caused by these closely related alphaherpesviruses in ocular, oral and genital mucosae.

### 5.1. Ocular Herpes Immunopathology

Recurrent HSV-1 infections can induce herpetic stromal keratitis (HSK), a blinding immunopathologic disease characterized by progressive scarring in corneal stroma [206]. In mice, HSV-1 ocular infection induces infiltration of inflammatory cells in two major waves. The first and transient wave of polymorphonuclear cells (PMNs) infiltration dissipates within 4 days after infection [207]. This PMN infiltration controls HSV-1 replication, which coincides with the appearance of corneal epithelial lesions. These epithelial lesions heal by day 4 after infection and the corneas appear normal by both clinical and histopathological exams. This PMN infiltration appears to be T-cell independent as it occurs in both normal and T-cell-deficient mice [207, 208]. After viral clearance from the cornea, a second wave of a chronic leukocytic infiltration is initiated between day 7 and 10 after infection. This secondary infiltrate consists of neutrophils, CD4^+^ T cells, few CD8^+^ T cells, dendritic cells (DCs), and macrophages [208–212]. Unlike the first wave, the second wave of cell infiltration seems to be T-cell-dependent and is orchestrated by CD4+ T cells [209, 210, 213, 214]. Indeed, ocular HSV-1 infection of athymic nude mice failed to cause HSK. However, T cell-deficient mice develop HSK following adoptive transfer of exogenous HSV-specific T cells [213, 214]. 

 The involvement of CD4^+^ T cells that produce Th_1_ cytokines (IL-2 and IFN-*γ*) in HSK has been established in the mouse model, and HSK can be abrogated by depletion of CD4^+^ T cells or neutralization of Th_1_ cytokines [215–220]. The susceptibility to HSK in vulnerable A/J mice is not associated with the magnitude of the systemic HSV-specific CD4^+^ T-cell response generated in the draining lymph nodes (DLN) [221]. HSK is rather associated with the nature of CD4^+^ T cells (i.e., Th_1_ versus Th_2_), which can regulate inflammatory responses. Th_1_ type cytokines, such as IL-2 and IFN-*γ*, play an essential role in regulating neutrophil infiltration in the cornea, and both have been implicated in HSK. The role of CD4+ T cells producing Th_2_ cytokines in HSK is still controversial. Jayaraman et al. showed that adoptive transfer of gD_5-23_-specific Th_2_ cells into susceptible mice increased both the onset and severity of HSK after corneal HSV-1-infection [222]. Others suggest that HSK severity is ameliorated by CD4+ T cells expressing the Th_2_ cytokine IL-4 [223, 224]. The effect of CD4+ Th_2_ T cells on HSK might also be affected by the plasticity of these cells. The susceptibility to HSK is also determined by the capacity of HSV-specific CD4^+^ T cells to induce DCs and neutrophils infiltration into the cornea. A recent study by Divito and Hendricks demonstrated that HSV-1-infected corneas without HSK contained similar numbers of activated CD4^+^ T cells as detected in HSV-1 infected corneas with maximal HSK severity [225]. In humans, activated HSV-specific T cells, infiltrate patient's cornea with HSK [226–228]. CD4^+^ Th_17_ T cells have also been implicated in human HSK [229]. Our preliminary data revealed that following ocular HSV-1 infection, the corneas of susceptible HLA Tg mice appear to be infiltrated with CD4^+^ T cells. However, the relative contribution of cornea-resident effector memory T cells (T_EM_) and DLN-resident central memory T cells (T_CM_) in protective memory against genital herpes versus immunopathology remains to be determined.

### 5.2. Genitomucosal Herpes Immunopathology

Shedding of reactivated HSV is estimated to occur at rates of 3–28% in seropositive adults who harbor latent HSV-2 in their sensory ganglia [36, 230–232]. However, the vast majority of these immunocompetent individuals do not experience recurrent herpetic disease. These are “asymptomatic patients” [13, 36, 112, 233]. In contrast, in some immunocompetent individuals, reactivation of latent virus leads to recurrent disease [13, 36, 112]. Recurrent disease ranges from rare episodes occurring once every 5–10 years to outbreaks occurring monthly or even more frequently among a small proportion of subjects [13, 234, 235]. Individuals with a well-documented clinical history of at least 5 recurrent genital disease episodes during the past 12 months are “symptomatic patients.” The difference between “symptomatic” and “asymptomatic” genital herpes patients is not due to differences in virus reactivation rates, since both groups have similar virus-shedding rates [36]. Patients with T-cell immune deficiencies, whether genetic or acquired, appear to suffer more frequent reactivation and greater disease severity than immunocompetent persons [37]. This emphasizes the crucial role of cell-mediated immunity [145, 146]. The most important T-cell epitopes may be in the tegument proteins, which are injected into the cell during viral entry, including those encoded by genes UL25 and UL39 [236] and envelope glycoproteins (i.e., gB and gD) [1, 153, 237]. Substantial research has been directed towards the development of T-cell epitope-mediated vaccines that are based on the identification and inclusion of immunogenic T-cell epitopes. However, not all immunogenic T cell epitopes are protective in nature, and some may even be harmful [238]. A good starting point for the development of an effective herpes vaccine would be to identify T-cell epitopes from HSV envelope and tegument proteins that are recognized by “asymptomatic” patients, to include these “protective” epitopes in the vaccine, and to exclude the symptomatic epitopes that might be “pathogenic” and harmful. Vaccines excluding (pathogenic) “symptomatic” epitopes should have increased efficacy against disease. 

Several lines of evidence, in both animal models and humans, support a critical role for T cells in controlling genital herpes infections and disease [136, 153]. The precursor frequency of HSV-2-specific CD8^+^ CTL is correlated with HSV-2 disease severity in HIV-1/HSV-2 coinfected humans [239]. HSV-2-specific CD4^+^ and CD8^+^ T cells persistently infiltrate healed genital herpes lesions [153]. In mice, following ocular infection, the HSV-1-specific CD8^+^ T cells infiltrate the infected ganglia during both acute and latent phases and may mediate control of viral reactivation in an IFN-*γ*-dependent manner [136]. In human genital biopsy specimens with recurrent HSV-2 disease, viral clearance is associated with a high concentration of local virus-specific CD8^+^ CTLs [240]. Considering the wealth of information addressing the role of T cells in animal models, it is surprising how few reports exist exploring the immunologic basis of symptomatic and asymptomatic HSV infections in humans. Thus, we recently detected such segregation of human CD4^+^ T-cell epitopes in gB [1].

 The outcome mother-to-infant HSV-2 vertical transmission and neonatal infection is orchestrated by two main factors: (1) the virus itself, which can directly cause the injury and (2) the maternal and fetal immune responses, which either protect from or exacerbate the neonatal disease. Particularly, cell-mediated immune responses can be a double-edged sword during pregnancy: (a) cellular immunity is important in viral control and clearance of infected tissues; (b) an overactive cellular immunity within the delicate environment of the placenta could also cause immunopathology. The mother's immune response, while necessary to reduce viral burden in the placenta, may also result in cell-mediated pathology in the placenta and the fetus, leading to placental/fetal dysfunction, fetal injury, fetal sequelae, and/or potentially the loss of the fetus. Regrettably, there is little known about whether and how maternal T cells responses affects neonatal herpes infection and what consequences this has for the newborn. 

## 6. Concluding Remarks

- HSV-1 and HSV-2 are infectious pathogens that cause serious diseases at every stage of life, from fatal disseminated disease in newborns to cold sores, genital ulcerations, eye disease, and fatal encephalitis in adults. Mucosal surfaces constitute an impressive first-line defense that is frequently exposed to HSV-1 and HSV-2 infections [80, 241–243].
- Most of the current drug therapies are either ineffective or inadequate for preventing the mucosal surface from the invading HSV-1 and HSV-2. Among the tools available for disease prevention and control, vaccines rank highly with respect to effectiveness as well as logistic and economic feasibility.
- Identifying the cellular and molecular immune composition of the various mucosal immune systems that are exposed to herpes infection should lead to an improved understanding of the immune-mediated herpetic infectious diseases and tissue destruction during various inflammatory states. This should be helpful in developing safe mucosal immunoprophylactic and/or immunotherapeutic vaccine strategies.
- An important lesson learned from the preclinical and clinical vaccine trials described above is the true feasibility (i.e., practicability) of a herpes vaccine. “Common denominators” between most of the above vaccine strategies are that (i) the cellular immunity appears to be crucial, rather than the humoral immunity, in protecting against herpes; (ii) the delivered antigens includes both “symptomatic” and “asymptomatic” T-cell epitopes.
- Considering the limited success of the recent herpes clinical vaccine trial [5], new mucosal vaccine strategies are needed.Our recent findings show that T cells from symptomatic and asymptomatic men and women (i.e., those with and without recurrences, resp.) recognize different herpes epitopes. Mucosal immunization with HSV-1 and HSV-2 epitopes that induce strong in vitro CD4 and CD8 T-cell responses from PBMC derived from asymptomatic men and women (designated here as “asymptomatic” protective epitopes”) could boost local and systemic “natural” protective immunity induced by wild-type infection.
- Mucosal subunit vaccines are designed for needle-free application, therefore safe and cost effective compared to other vaccines. Tremendous research efforts have significantly improved the classical approach used to create these vaccines and alternative methods of immunization based on new concepts of mucosal immunity are being developed.
- HSV-specific CD8^+^ T cells, selectively activated and retained in latently infected sensory ganglia [136, 153, 244, 245], play a crucial role in suppressing full-blown reactivation [136, 246] by interfering with virus replication and spread. Thus, rather than completely eliminating the latent HSV-1 from sensory ganglia, reactivations appear to be “kept in check” by CD8^+^ T cells [138, 139, 153, 247].
- It is still unclear why and how the virus manages to sporadically escape CD8^+^ T-cell-mediated immunosurveillance and reactivate from latency, causing ocular and genital herpes diseases. Identification of the immune evasion mechanisms would help develop stronger preemptive immunotherapeutic vaccine strategies against herpes.
- We believe that future research endeavors should focus on (1) identifying more “asymptomatic” versus “symptomatic” herpes epitopes, (2) qualitatively and quantitatively analyzing T cells in symptomatic versus asymptomatic patients to understanding the immune mechanisms underlying herpes pathogenesis in humans, (3) incorporating only promiscuous “asymptomatic” epitopes into vaccines, (4) using mucosal vaccine strategies, such as lipopeptides, to immunize against herpes, and (5) using “humanized” susceptible HLA transgenic mice and rabbits to assess the immunogenicity and protective efficacy of herpes epitopes against primary and recurrent infection.
- A targeted mucosal immunotherapeutic vaccine is necessary to induce robust localized immune responses (i.e., in the central nervous system, trigeminal ganglia, and sacral ganglia), to quell virus replication, to drive the pathogen into a "latent" state, and likely hinder reactivation.
